# Mental Health in Community Colleges: A Review

**DOI:** 10.1007/s11920-026-01668-2

**Published:** 2026-02-21

**Authors:** Jordan Rasp, Meera Menon

**Affiliations:** The Ohio State University Wexner Medical Center, 1670 Upham Dr, Columbus, OH 43210 USA

**Keywords:** Community college, Two-year college, College mental health, Campus mental health services, Access to care, Psychiatry, Young adults

## Abstract

**Purpose of Review:**

This paper examines the various perceptions of community college students on mental health challenges, access to resources, barriers to treatment, and possible interventions for care improvements. Data was gathered via literature review.

**Recent Findings:**

Resources for college students enrolled in a two-year or community college program have been limited despite similar or higher rates of mental health crises compared to their four-year student counterparts.

**Summary:**

Overall, these findings point to the necessity of advancing initiatives to close the mental health treatment gap between community colleges and four-year universities, as well as between racial and ethnic minority students within community colleges. A better understanding of how mental health affects community college students could inform more effective interventions from college staff and clinicians. More research is needed to generalize results and implement interventions to improve access to care.

## Introduction

Community colleges offer numerous opportunities for learners to advance their education, develop skilled trades and marketable skills aligned with local workforce needs, access geographically convenient learning environments, receive support for employment within their chosen fields, and benefit from lower costs compared to traditional four-year college programs. While mental health challenges among college students have been well studied, there are few studies specifically examining students engaged in two-year or community college programs. This subset of college students encompasses a larger proportion of individuals from racial and ethnic minority groups, those from lower socioeconomic classes, single parents, first-generation college students, older students, and those who are more likely to be working in addition to attending classes [1]; all of which have potential impacts on students’ mental health. Despite studies suggesting that students attending two-year programs may face mental health challenges more frequently relative to four-year university students, there is also a concern for inadequate resources in this student population subset [1]. In fact, the ratio of counselors to students at community colleges was found to be one counselor per 3000 students, significantly less than their four-year counterparts, who, on average, have one counselor per 1600 students [2].

According to the American College Counseling Association’s 2023 survey collected from over 200 different community college programs, only 16% of college programs have a psychiatrist or licensed prescriber on staff [3]. While the number of colleges having some sort of counseling programming for mental health has been increasing over the years, 53% of survey respondents are somewhat or not-at-all satisfied with how their college responds to at-risk students and crises [3].

## Prevalence of Mental Illness Among Community College Students

Much of the mental health research across the college student population is based on research conducted at four-year universities. However, it is important to consider the impact of mental health on community college students as well, given that 40% of the undergraduate student population is attending a two-year or community college program [1]. Few studies have specifically focused on research targeting community college students.

One study by Eisenberg et al., partnered with Health Minds Network at the University of Michigan, the Wisconsin HOPE Lab at the University of Wisconsin-Madison, the Association of Community Colleges, and Single Stop, conducted an online survey across 10 community colleges [2]. The survey was used to examine the prevalence of mental health on campus and how students were using mental health services. Students were chosen to participate at random and completed a modified version of the Healthy Minds Study. The survey measured recent symptoms of depression using the PHQ-9 screening tool, anxiety using the GAD-7 screening tool, and eating disorders using a well-studied SCOFF assessment. Additionally, the survey measured suicidal ideation and non-suicidal self-injury with three questions adapted from the National Comorbidity Survey Replication [2]. The survey also examined the utilization of mental health services, including whether students were prescribed medications and/or received counseling.

The results demonstrated that 49% of community college students reported at least one mental health condition [2]. Community college students under the age of 25 were more likely to report a mental health condition, with a prevalence of 56% in comparison to a prevalence of 42% among older students [2]. The results from the survey were compared to students who participated in the Healthy Minds Study in 2014–2015 from four-year programs. Among four-year university students aged 25 or younger, only 46% reported a mental health condition, representing a 10% lower rate compared to their community college student counterparts. This difference was especially notable for younger students endorsing symptoms of depression, as 23% of community college students reported symptoms of depression compared to 11% of four-year students of the same age group [2].

Although 49% of community college students identified a mental health condition, only 41% indicated they had accessed mental health services within the past year. This rate was slightly lower than that of four-year university students (45.7%) [2]. Of note, community college students had higher rates of being uninsured (13.5%) compared to four-year students (3.1%), likely impacting their options for support [2]. A limitation of this study was the small response rate of 9%. There was concern for bias either in over-sampling students struggling with mental health, as they may have been more interested in completing the survey, or in the opposite direction, as students struggling with mental health may be less motivated to participate [2].

A more recently published study by Lipson et al. analyzed data from the 2016–2019 Healthy Minds Study, including 10,089 students from 23 community colleges and 95,711 students from 133 four-year institutions [4]. The prevalence of students meeting criteria for one or more mental health problems remained high, with over 50% of students at both community and four-year institutions meeting criteria. Notably, the highest prevalence of mental health concerns continued to be observed among younger students attending community colleges, compared to same-age peers at four-year institutions [4]. Differences in the use of mental health services remained significant, with community college students accessing therapy resources at lower rates (30%) compared to students at four-year institutions (39%), particularly among those community college students aged 18–22 years (25.3%) [4]. Barriers to treatment also differed between community college students and those attending four-year institutions. Financial constraints were the most common barrier among community college students, whereas students at four-year institutions cited time constraints as their biggest barrier [4].

Another study by Cadigan et al., surveyed over 900 community college students in the Pacific Northwest and asked them to identify major health concerns impacting community college students [1]. Participants were recruited from three public community colleges via posters, handouts on campus, advertisements, and emails. The study used results from a brief eligibility survey that students completed for a chance to participate in a larger study and win $250. In the survey, participants were asked to answer questions about their demographics, alcohol and marijuana use, mental and physical health, and to “list three health issues facing community college students today [1]. ”

The study found that a little less than a third of students (28.5%) rated their mental health as “fair” or “poor” over the last year [1]. When all students were asked to identify three health issues facing community college students, 60% of students listed a mental health issue. The students who rated their own mental health as “fair” or “poor” were found to be more likely to identify either a mental health concern or an access to care issue as one of the three health issues facing students today [1]. When considering demographics, women were statistically more likely to identify a mental health issue compared to men; no significant sex differences were identified when listing other health issues facing community college students, such as medical issues, substance use, or access to care issues [1]. A limitation of this study was that the extent to which the identified health issues were relevant to the participant’s personal life was unknown. However, given the frequency with which a mental health issue was listed as one of the major health concerns, one could conclude that mental health concerns have a large direct or indirect impact on campus [1].

### Impact of Identity on Mental Health Symptoms and Help Seeking in Community Colleges

Community colleges often yield a uniquely diverse student population with a larger percentage of students from racial and ethnic minority groups, lower-income backgrounds, older students, first-generation college students, and students who may be balancing significant responsibilities outside of school, such as full-time jobs or single parenting [1]. Research has shown that students who work over 20 h a week are at greater risk of not completing their education [5]. Looking at the 2019–2020 academic year for community college students enrolled full-time, 43% worked full-time jobs and 30% were employed part-time [6]. Compared to full-time four-year university students, 40% of students had a job, but only around 10% worked over 35 h a week [7]. In a study by Peltz et al., researchers surveyed 792 undergraduate students in New York to examine the relationship between sleep, depression, and employment hours [8]. The study found that in students from lower socioeconomic backgrounds, increased work hours indirectly predicted higher levels of depression via sleep disturbances as a mediator [8].

Approximately 53% of undergraduates at two-year, public institutions were racial/ethnic minority students, compared to 45% of their four-year counterparts [9]. In a study by Lipson et al., investigators gathered data from the national Healthy Minds Study from 2013 to 2021 to examine the impact of holding an ethnic or minoritized identity in regards to mental illness and seeking care. This study is notable as it was the first multi-campus, nationwide study examining mental health trends in minority undergraduate students across time. Results found that the prevalence of symptoms of depression, anxiety, suicidal ideation, or having one or more mental health problems increased over time across all ethnic groups, but most significantly in racial/ethnic minority students [9]. This increase in prevalence was seen most significantly in students identifying as American Indian/Alaskan Native [9]. Despite this increasing prevalence, the study found continued inequalities regarding mental health treatment in minority students. For example, while the prevalence of meeting criteria for one or more mental health problems rose 45% among multiracial students from 2013 to 2021, there was only a 9% increase in treatment for these students [9]. These findings highlight the need for continued efforts to address the mental health treatment gap among racial and ethnic minority students.

The 2023 Community College Survey of Student Engagement (CCSSE) included 61,085 students from 149 community colleges and provided further insight into the rising mental health challenges among community college students [10]. One-third of respondents reported not knowing where to seek mental health treatment, a gap that was particularly pronounced among Hispanic or Latino students. Among those who reported needing mental health support in the past year, 42% never sought help, with Hispanic or Latino students experiencing this at higher rates. A lack of available resources was identified as the most significant barrier to seeking care [10].

## Use of Mobile Applications Among Community College Students

In a study by Borghouts et al., investigators explored the use of mental health mobile apps to help mitigate the limited access to resources that community college students often experience compared to four-year university students [11]. Investigators used email addresses from the community college’s registrar’s office to invite 5000 students at random to participate. The online survey took around twenty to thirty minutes to complete, and participants received a $10 gift card as an incentive for their participation. The response rate was about 11.5% and 500 students completed the survey. From the survey, participants identified mental health barriers, how COVID-19 impacted their lives, their experience with a mental health crisis, whether they use/used or were interested in apps for mental health, past use of mental health services, perceived stress, if they’ve ever felt they needed to see a professional for their mental health, mental health concerns over the past year, perceived stigma, how friends and family felt about them using a mental health app, and privacy concerns [11].

The study revealed that 37.8% of participants reported experiencing a mental health condition, and a little under half of those participants felt their mental health condition was related to stress [11]. 21.2% of participants reported having previously used a mental health app, while an additional 39.8% expressed interest in using one despite never having done so before [11]. The most important aspect for participants in using a mental health app was that the app was free. Participants’ privacy was additionally of utmost importance. The most significant factors positively associated with a participant’s use of a mental health app included perceived stress and need to seek help, past mental health treatment, and others’ influence. The concern for privacy was negatively associated with app use [11]. There were several limitations in this study, but notably, participants were from a single community college, which may limit its generalizability. The survey was also distributed one month into the COVID-19 pandemic during the mandatory stay-at-home order, making it hard to know whether the results were reflecting typical patterns or pandemic-specific effects [11].

It is imperative that apps advertised to improve mental health are evidence-based, as non-validated tools may potentially worsen symptoms or provide misleading advice. An increasing number of individuals are turning to artificial intelligence (AI) to treat their mental health concerns. Community college students, particularly those from minoritized or lower socioeconomic backgrounds who may face barriers to accessing mental health care, may be especially vulnerable to reliance on AI-based alternatives or non-evidence-based apps. Risks associated with primarily AI-based tools, such as ChatGPT, include a lack of individualized support during mental health crises, absence of human interaction, empathy, and clinical judgment, and the potential for biased responses based on historical data, which may result in harmful misunderstandings or advice [12].

## Increasing Access to Mental Health Care

The California Community College (CCC) network serves over two million students across 116 campuses in the state of California [13]. To help address the high demand for mental health services, the CCC provides six to eight counseling sessions per academic term. Students needing more sessions are referred to providers in the community. However, this often creates fragments in care due to students having difficulty navigating the referral system, insurance, and wait times. A pilot program conducted by Trieu et al., was completed across four community colleges in San Francisco, California, during the 2020–2021 academic year in an attempt to improve community college students’ access to mental health care [13]. Students interested in a career in social work, nursing, and social sciences were recruited as “mental health navigators” to assist peers in connecting with mental health services within the community. Navigators were provided education on mental health topics in the fall semester, and hands-on learning occurred in the spring. The pilot program aimed to increase access to mental health services, but also hoped for success with the intervention, given past literature noting improvements to care using similar peer models [13].

Regarding the process of the program, the navigators were connected to students receiving mental health counseling near the end of their six to eight provided sessions via email. The navigators primarily worked with students with already existing health insurance to find providers accepting their insurance in the community. As linkage could take three to five weeks, the navigators checked in with the students weekly to seek updates on their progress in transitioning care. After the students were linked in the community, the navigators checked in one week and one month after their first appointment to follow up on their progress and perform a quality-of-care survey [13].

As a result of the pilot program, 58% of students were successfully connected to a community mental health agency. The navigators found the program beneficial as it exposed them to the mental health field and enhanced their career interests. One challenge noted by authors was that students requiring mental health support were recruited for the pilot program in the summer when student enrollment was the lowest. The attrition rate of navigators was also moderate, as two of the ten navigators did not complete the program. Authors also noted that more training and practice sessions may help navigators feel more prepared in the future to implement referral strategies. Other future goals include improving the relationship with the navigators and community providers and sharing the program with other community colleges to test its use in other student populations [13].

## Conclusion

Community college students constitute a diverse and increasingly significant sector of the undergraduate population. Current evidence suggests the prevalence of mental health conditions amongst community college students as shown to be at or above the rate of their four-year counterparts. As mental health concerns continue to rise, it is imperative to continue exploring strategies to increase access to resources and address the disparities in care within community college settings. Innovative interventions, like the mobile app or mental health navigators, should continue to be explored and show promise in expanding access to care. More studies focused specifically on community college populations are essential to understand the impact of mental health challenges and to guide interventions that can be generalized across community college campuses.

## Key References

- Cadigan JM, Duckworth JC, Lee CM. Physical and mental health issues facing community college students. J Am Coll Health. 2022 Apr;70(3):891-897. doi: 10.1080/07448481.2020.1776716. Epub 2020 Jun 22. PMID: 32569500.
  - ○ This reference is particularly significant because it provides essential background information on the demographics of community college students and the effects of mental health on this population.

- Lipson SK, Zhou S, Abelson S, Heinze J, Jirsa M, Morigney J, Patterson A, Singh M, Eisenberg D. Trends in college student mental health and help-seeking by race/ethnicity: Findings from the national healthy minds study, 2013-2021. J Affect Disord. 2022 Jun 1;306:138-147. doi: 10.1016/j.jad.2022.03.038. Epub 2022 Mar 18. PMID: 35307411; PMCID: PMC8995361.
  - ○ This study demonstrates the rising prevalence of mental health concerns among minoritized students and emphasizes the importance of sustained efforts to close the treatment gap for racially and ethnically minoritized students.

- Trieu SL, Chen R. Community College Mental Health Navigators: A Pilot Program to Improve Access to Care. Health Promot Pract. 2023 Nov;24(6):1138-1141. doi: 10.1177/15248399221090917. Epub 2022 May 4. PMID: 35506378. 
  - ○ This study illustrates a pilot program that can be effectively implemented on community college campuses to enhance access to mental health care and highlights the need for continued development of similar innovative approaches to support community college students.

## Data Availability

No datasets were generated or analysed during the current study.
